# Simultaneous clavicular hook plate fixation in bilateral distal clavicle fractures (Neer type II): A case report

**DOI:** 10.1097/MD.0000000000040398

**Published:** 2024-11-01

**Authors:** Bedrettin Akar, Fatih Ugur

**Affiliations:** a Department of Orthopedics and Traumatology, Sakarya Yenikent State Hospital, Sakarya, Turkey; b Department of Orthopedics and Traumatology, Kastamonu University Faculty of Medicine, Kastamonu, Turkey.

**Keywords:** accident, acromioclavicular joint, bone fractures, clavicle, osteosynthesis

## Abstract

**Rationale:**

Bilateral distal clavicle fractures (BDCF) are exceedingly rare types of fractures. This study aimed to evaluate the surgical treatment under a single anesthesia for trauma-induced BDCF (Neer type II) through a case report.

**Patient concerns:**

The patient brought to the emergency department due to a motor vehicle accident exhibited severe pain, tenderness, swelling, and deformity in both shoulder regions upon physical examination. No significant pain or tenderness was detected in other areas of the body. There were also no signs of additional neurological deficits or vascular pathology in the extremities.

**Diagnoses:**

Radiographic examinations led to a diagnosis of BDCF (Neer type II).

**Interventions:**

Surgical intervention involved the simultaneous application of a neutral-angled hook plate to both clavicles via open reduction.

**Outcomes:**

Postoperative radiographs taken at the 12th week demonstrated complete healing in both fractures and no functional limitations in shoulder movements.

**Lessons:**

BDCF can compromise the stability of the shoulder girdle, necessitating surgical intervention with anatomical reduction and rigid fixation.

## 1. Introduction

Clavicular fractures represent 3% to 5% of adult fractures and 32% of shoulder girdle fractures. The middle third, being the thinnest part of the clavicle, is where most fractures occur.^[1,2]^ The clavicle is directly connected to 2 joints, and nonunions can lead to biomechanical disruptions in the shoulder girdle. The medial third of the clavicle is in close proximity to important neurovascular structures, such as subclavian vascular structures and the brachial plexus, while the distal third contributes to stability through the acromioclavicular and coracoclavicular ligaments.^[2–4]^ Clavicular fractures can result from direct or indirect trauma. Various factors, such as sports injuries, motor vehicle accidents, and falls, can contribute to the etiology of these fractures. The mechanism of injury often involves a fall onto the shoulder when the arm is abducted.

The classification of clavicular fractures is based on anatomical location, displacement degree, and ligament status. The Allman classification is commonly used today, dividing clavicular fractures into 3 groups: middle third (75%), proximal third (3%), and distal third (22%). Distal clavicular fractures are further classified by the Neer classification into 3 types.^[4,5]^ In type I, the fracture is distal to the coracoclavicular ligaments (trapezoid-conoid) with minimal displacement since the ligaments remain intact, preserving the acromioclavicular joint. Conservative treatment is typically recommended for these fractures. In type II, the medial fragment is detached from the fracture, leaving the lateral fragment in place, while the medial fragment undergoes displacement. Internal fixation is generally recommended for type II fractures. Distal clavicular fractures of Neer type II can be further classified into 2 subgroups based on the integrity of the conoid and trapezoid ligaments. Both the conoid and trapezoid ligaments remain attached to the distal fragment in type IIA, while the trapezoid ligament remains attached to the distal fragment, while the conoid ligament is detached in type IIB. Therefore, type IIB carries a risk of displacement and nonunion due to the absence of contact between the medial portion of the coracoclavicular ligament. In type III fractures, the fracture extends distally to the coracoclavicular ligaments and reaches the acromioclavicular joint. The problem with these fractures is the potential development of arthrosis or osteolysis in the acromioclavicular joint in later stages.^[5,6]^ Clinical manifestations of clavicular fractures commonly include anterior shoulder pain, swelling, edema, and crepitus at the trauma site.

Although rare, neurovascular injuries can occur in clavicular fractures either during or following the traumatic event or during surgery.^[7]^ While conservative management is generally the preferred treatment approach, surgical options are typically considered for cases exhibiting significant displacement or shortening exceeding 2 cm.^[6–8]^ This study aimed to discuss the surgical treatment under a single anesthesia of trauma-induced bilateral distal clavicle fractures (BDCF) (Neer type IIB) through a case report.

## 2. Case report

**Figure 1.**
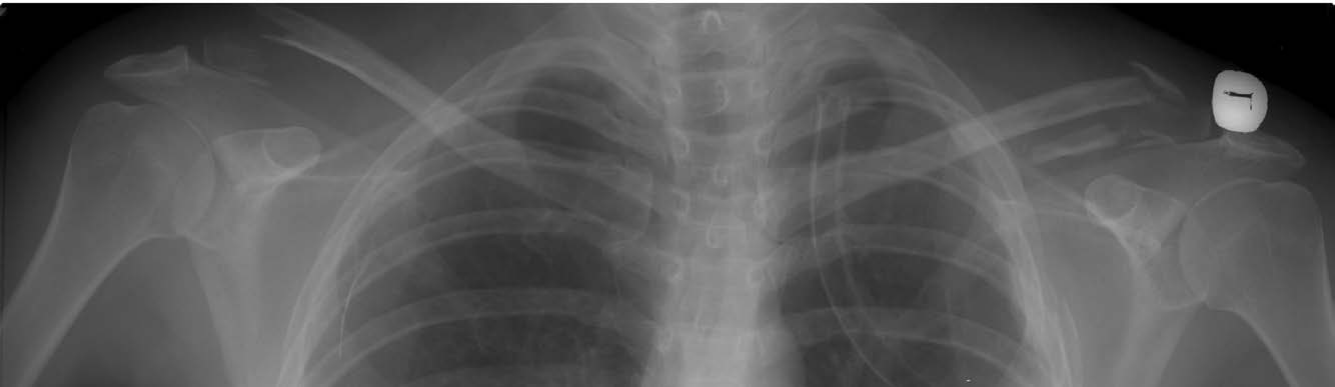
Preoperative bilateral shoulder radiography image.

A 42-year-old female patient presented to the emergency department following a motor vehicle accident. On physical examination, the patient exhibited severe pain, tenderness, deformity, and swelling in both shoulder regions. Shoulder anteroposterior radiographs and computed tomography scans revealed BDCF (Neer type IIB) (Fig. 1).

**Figure 2.**
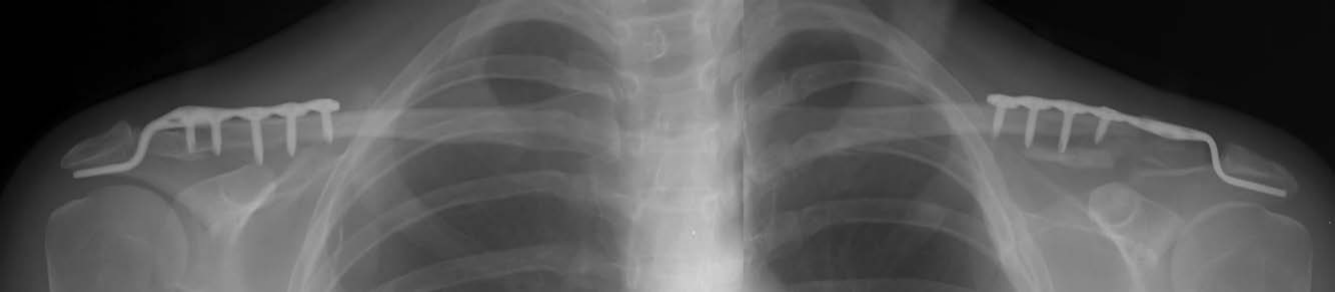
Postoperative early-period radiography (first month).

**Figure 3.**
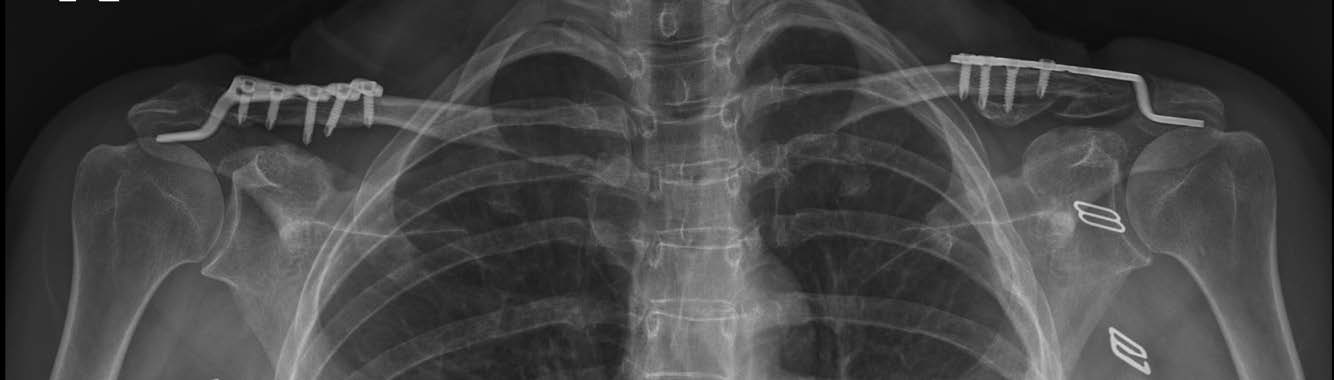
Postoperative late-period radiography (fifth year).

**Figure 4.**
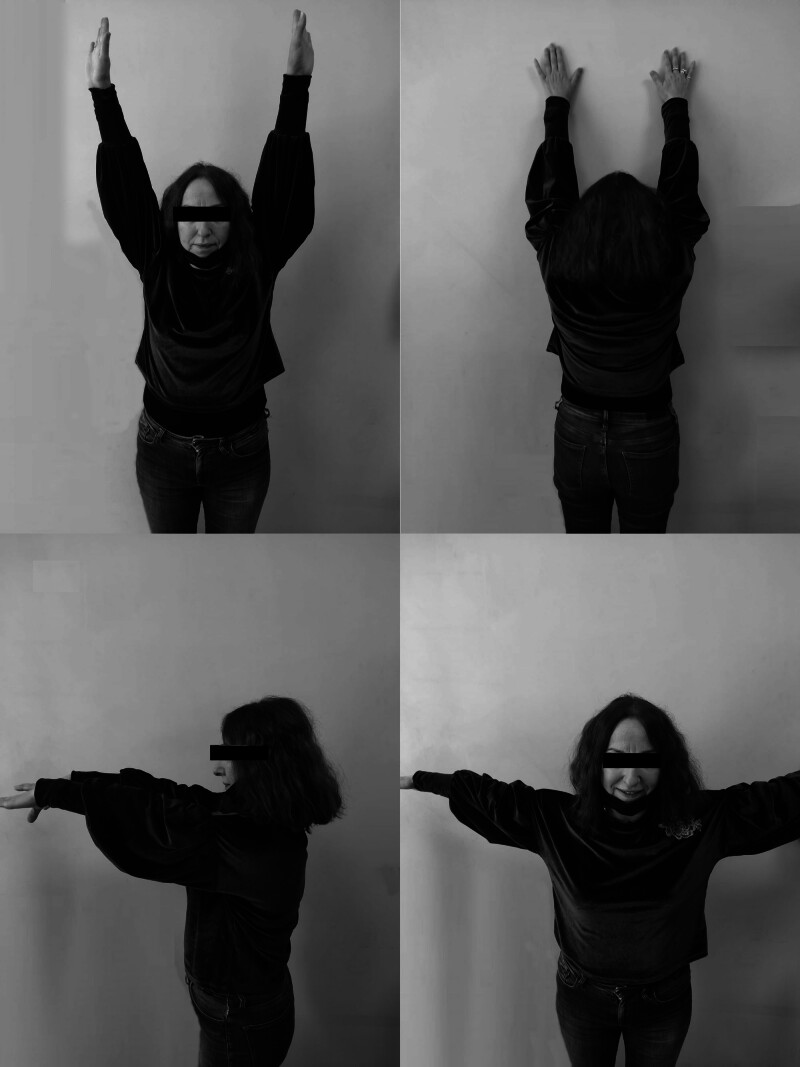
Patient’s fifth-year shoulder functions.

Apart from simple lacerations related to trauma, no additional fractures, neurological deficits, or vascular pathology were identified in the patient. Both shoulders were temporarily immobilized using Velpeau bandages. The patient had no comorbidities and was prepared for surgery to achieve anatomical reduction of both clavicles due to the advanced degree of displacement observed during radiographic evaluation. The surgical plan involved open reduction and internal fixation using neutral-angled clavicular hook plates, first on the right and then on the left clavicle. Prophylactic treatment included 1 g of cefazolin administered 3 times a day, 0.6 mL of enoxaparin once a day, and non-steroidal anti-inflammatory drugs. For the surgical technique, the patient was placed in the beach chair position under general anesthesia. A 10-cm incision was made approximately 2 cm behind the distal end of the clavicle, extending toward the medial aspect. This allowed clear visualization of the clavicle, acromioclavicular joint, and acromion. The fracture line was cleaned and reduced. Due to the insufficient size of the bone fragment in the clavicle, the neutral-angle clavicular hook plate was correctly positioned from the back of the acromioclavicular joint and adjusted to fit the smaller clavicle after the fracture was repositioned. After ensuring fracture stability and controlling bleeding, the layers were anatomically closed, followed by the application of Velpeau bandages to both shoulders. After 2 weeks, the bandages were removed, and active and passive exercises were initiated for rehabilitation. Postoperative radiographs taken at 4, 8, and 12 weeks revealed complete union of both fractures without any functional limitations in shoulder movements (Fig. 2). As there were no clinical or functional complications observed in radiographs taken during the fifth year (Figs. 3 and 4), and as per the patient’s preference, the implants were not removed.

## 3. Discussion

BDCF cases are extremely rare. Due to the high rates of complications associated with surgical intervention, clavicular fractures are generally treated using conservative methods.^[8]^ However, recent literature has reported an increase in cases of nonunion and shoulder dysfunction due to conservative treatment. Particularly in BDCF cases, conservative treatment should be avoided as the shoulder girdle becomes unstable.^[8,9]^ In such fractures, aiming for anatomical reduction for both clavicles and primarily considering surgical intervention are essential to ensure shoulder stabilization and achieve optimal functional outcomes.

Zhang et al^[10]^ applied a hook plate to a 16-year-old patient with BDCF (Neer type II) and emphasized the importance of shoulder stabilization. They achieved excellent results in shoulder movements after a 2-year follow-up.^[10]^ Qi et al^[11]^ reported successful outcomes with reconstruction plate application for bilateral middle-third clavicle fractures. Sambandam et al^[12]^ noted a lack of consensus in the literature regarding BDCF treatment, with some surgeons opting for conservative management and others preferring surgery. In their study, they stated that surgical fixation was necessary for distal end fractures due to their unstable nature and helped prevent deformative forces affecting the fragments.^[12]^ In another study, Kim et al^[5]^ highlighted the absence of a consensus despite numerous treatment options for BDCF. They emphasized that conservative treatments could lead to complications such as nonunion and pseudoarthrosis, making surgical fixation preferable to achieve stable fixation.^[5]^ Wang et al^[13]^ reported that using titanium cables and locking plates in Neer type II distal clavicle fractures resulted in highly successful outcomes with low complication rates. They also noted that the use of hook plates could cause subacromial osteoarthritis, potentially impacting shoulder functions negatively.^[13]^ Finally, Erdle et al^[14]^ compared locking and hook plates in the treatment of Neer type II fractures, concluding that both implants were effective in fracture union with no significant difference between them.

## 4. Conclusion

BDCF, particularly Neer type II fractures, are highly uncommon pathologies typically associated with major trauma etiologies. Due to their rarity and the potential for being mistaken for unilateral fractures in emergency settings, a correct diagnosis requires a thorough clinical history, a detailed physical examination, and adequate imaging. In terms of treatment, we consider that surgical planning aiming for anatomical reduction and rigid fixation would be the appropriate choice, given that bilateral fractures destabilize the shoulder girdle, limiting the efficacy of conservative options.

## Author contributions

**Conceptualization, resources, writing – original draft, writing – review & editing:** Bedrettin Akar, Fatih Ugur.

**Investigation, methodology:** Bedrettin Akar.
